# The effect of water immersion and acute hypercapnia on ventilatory sensitivity and cerebrovascular reactivity

**DOI:** 10.14814/phy2.13901

**Published:** 2018-10-18

**Authors:** James R. Sackett, Zachary J. Schlader, Carol Cruz, David Hostler, Blair D. Johnson

**Affiliations:** ^1^ Center for Research and Education in Special Environments Department of Exercise and Nutrition Sciences University at Buffalo Buffalo New York

**Keywords:** Central chemoreceptors, elevated CO_2_ pressure, head out water immersion, hemodynamics, ventilation

## Abstract

The partial pressure of end tidal carbon dioxide (PETCO_2_), ventilatory sensitivity to CO_2_, and cerebral perfusion are augmented during thermoneutral head out water immersion (HOWI). We tested the hypotheses that HOWI and acute hypercapnia augments minute ventilation, ventilatory sensitivity to CO_2_, cerebral perfusion, and cerebrovascular reactivity to CO_2_. Twelve subjects (age: 24 ± 3 years, BMI: 25.3 ± 2.9 kg/m^2^, 6 women) participated in two experimental visits: a HOWI visit (HOWI) and a matched hypercapnia visit (Dry + CO_2_). A rebreathing test was conducted at baseline, 10, 30, 60 min, and post HOWI and Dry + CO_2_. PETCO_2_, minute ventilation, expired gases, blood pressure, heart rate, and middle cerebral artery blood velocity were recorded continuously. PETCO_2_ increased throughout HOWI (baseline: 42 ± 2 mmHg; maximum at 10 min: 44 ± 2 mmHg, *P* ≤ 0.013) and Dry + CO_2_ (baseline: 42 ± 2 mmHg; maximum at 10 min: 44 ± 2 mmHg, *P* ≤ 0.013) and was matched between conditions (condition main effect: *P* = 0.494). Minute ventilation was lower during HOWI versus Dry + CO_2_ (maximum difference at 60 min: 13.2 ± 1.9 vs. 16.2 ± 2.7 L/min, *P* < 0.001). Ventilatory sensitivity to CO_2_ and middle cerebral artery blood velocity were greater during HOWI versus Dry + CO_2_ (maximum difference at 10 min: 2.60 ± 1.09 vs. 2.20 ± 1.05 L/min/mmHg, *P* < 0.001, and 63 ± 18 vs. 53 ± 14 cm/sec, *P* < 0.001 respectively). Cerebrovascular reactivity to CO_2_ decreased throughout HOWI and Dry + CO_2_ and was not different between conditions (condition main effect: *P* = 0.777). These data indicate that acute hypercapnia, matched to what occurs during HOWI, augments minute ventilation but not ventilatory sensitivity to CO_2_ or middle cerebral artery blood velocity despite an attenuated cerebrovascular reactivity to CO_2_.

## Introduction

Thermoneutral (~35°C) head out water immersion (HOWI) induces central hypervolemia, which causes several hemodynamic alterations, such as immediate increases in stroke volume, cardiac output, pulmonary blood flow, and cerebral blood flow (Arborelius et al. 1972; Farhi et al. 1977; Carter et al. 2014), while heart rate is slightly reduced (Farhi et al. 1977). Despite this, blood pressure does not change (Bonde‐Petersen et al. 1992; Sramek et al. 2000; Watenpaugh et al. 2000) or is slightly attenuated (Craig and Dvorak 1966; Sackett et al. 2017) during HOWI due to a reduction in total peripheral resistance. The hydrostatic pressure of the water increases the work of breathing (i.e., static lung load) (Lambertsen 1977; Moon et al. 2009), although minute ventilation does not change during HOWI compared to dry conditions (Sackett et al. 2017, 2018). Nevertheless, the partial pressure of end tidal carbon dioxide (PETCO_2_) is increased during HOWI (Sackett et al. 2017, 2018), which indicates carbon dioxide (CO_2_) retention (Lanphier and Bookspan 1999).

Typically, increases in arterial CO_2_ pressure stimulate the peripheral (Prabhakar and Peng 2004; Kumar and Prabhakar 2012) and central chemoreceptors (Nattie and Li 2012; MacKay et al. 2016) to increase ventilation in an attempt to lower arterial CO_2_ pressure. The absent increase in ventilation during HOWI, in the presence of elevated PETCO_2_, suggests that a small increase in PETCO_2_ is not sufficient to stimulate the chemoreceptors and cause a rise in ventilation. We have previously demonstrated that peripheral chemosensitivity to hypercapnia is not altered during thermoneutral HOWI (Sackett et al. 2017), whereas central chemosensitivity (i.e., ventilatory sensitivity to CO_2_) is augmented during thermoneutral HOWI (Sackett et al. 2018). Yet, it is not currently known if the increase in ventilatory sensitivity to CO_2_ during HOWI is due to the background of elevated PETCO_2_ alone or the combined effects of HOWI (i.e., elevated PETCO_2_, central hypervolemia, increased cerebral perfusion, increased work of breathing, etc.).

Increases in arterial CO_2_ pressure augment cerebral blood flow to enhance hydrogen “washout” at the site of the central chemoreceptors (Ainslie and Duffin 2009). The concomitant increase in PETCO_2_ and cerebral perfusion suggests that a link between the two could exist during HOWI (Carter et al. 2014). However, it is not clear if the rise in cerebral perfusion during HOWI is due to acute hypercapnia or the combined effects of HOWI, including elevated PETCO_2_. Furthermore, alterations in cerebral blood flow appear to modulate changes in cerebrovascular reactivity to CO_2_, such that increases in cerebral blood flow enhance cerebrovascular reactivity to CO_2_, while decreases in cerebral blood flow blunt cerebrovascular reactivity to CO_2_. Moreover, an attenuated cerebrovascular reactivity to CO_2_ enhances ventilatory sensitivity to CO_2_ (Fan et al. 1985; Xie et al. 2005; Ainslie et al. 2007), which indicates an important link between cerebrovascular reactivity to CO_2_ and ventilatory sensitivity to CO_2_. However, the effect of an augmented cerebrovascular reactivity to CO_2_ on ventilatory sensitivity to CO_2_ remains equivocal (Chapman et al. 1979). It is possible that an augmented cerebral blood flow, which occurs in the presence of elevated PETCO_2_ during HOWI, may enhance cerebral CO_2_ delivery and contribute to subsequent alterations in cerebrovascular reactivity to CO_2_ and ventilatory sensitivity to CO_2_. Yet, the independent effects of acute hypercapnia and HOWI on cerebrovascular reactivity to CO_2_ are not currently known.

The purpose of our study was to determine the effect of water immersion and acute hypercapnia on ventilatory sensitivity to CO_2_ and cerebrovascular reactivity to CO_2_. To test our hypotheses, we compared a HOWI visit (HOWI) to a dry condition where we matched the hypercapnia that occurred during HOWI (Dry + CO_2_). We hypothesized that: (1) minute ventilation and ventilatory sensitivity to CO_2_ are augmented during both HOWI and Dry + CO_2_ and (2) cerebral perfusion and cerebrovascular reactivity to CO_2_ are increased during both HOWI and Dry + CO_2_.

## Methods

### Subjects

Twelve subjects (age: 24 ± 3 years, BMI: 25.3 ± 2.9 kg/m^2^, 6 women) completed three visits: a screening visit and two experimental visits. Subjects reported to be recreationally active, nonsmokers, not taking medications, and free from any known cardiovascular, metabolic, neurological, or psychological disease. Women were not pregnant (confirmed via a urine pregnancy test). To control for menstrual cycle hormones, women were tested during the first 10 days following self‐identified menstruation (Minson et al. 2000). During the screening visit, subjects gave written consent following a comprehensive explanation of the experimental procedures and possible risks. The study was approved by the Institutional Review Board at the University at Buffalo and the study was performed in accordance with the standards set forth by the latest version of the Declaration of Helsinki.

### Instrumentation and measurements

Height and weight were measured with a stadiometer and scale (Sartorius Corp., Bohemia, NY, USA) and urine specific gravity was measured using a refractometer (Atago USA, Inc., Bellevue, WA, USA) prior to each experimental visit. Previous reports indicate that urine specific gravity is a valid indicator of hydration status (Armstrong et al. 1998). The CO_2_ pressure waveform was measured via a capnograph (Nonin Medical, Inc., Plymouth, MN, USA), of which the minimum value represented the inspired pressure of CO_2_ and the maximum value (i.e., end tidal) represented the expired pressure of CO_2_ (i.e., PETCO_2_). The sample rate of the capnograph was 4 Hz. PETCO_2_ was used as a marker of arterial CO_2_ pressure since it reflects arterial CO_2_ pressure during water immersion (Dunworth et al. 2017) and throughout a wide range of physiological dead space (McSwain et al. 2010), which may be increased during water immersion (Cherry et al. 2009). Inspired and expired ventilation was measured using a heated pneumotachometer (Hans Rudolph, Inc., Shawnee, KS, USA) that was attached to a silicone mouthpiece (Hans Rudolph, Inc., Shawnee, KS, USA). The fraction of expired CO_2_ was measured via a CO_2_ analyzer (Vacu Med, Venture, CA, USA), which was sampled at a 3 L mixing chamber. Beat to beat blood pressure was measured via photoplethysmography (ccNexfin Bmeye NA, St. Louis, MO, USA) on the left hand, which was suspended slightly above the water during HOWI. Blood pressure was corrected to the heart level using a height correction sensor. Heart rate was measured from a three‐lead electrocardiograph (DA100C, Biopac Systems, Inc., Goleta, CA, USA). Stroke volume was determined via the arterial pressure waveform using Modelflow (ccNexfin Bmeye NA, St. Louis, MO, USA) (Wesseling et al. 1993). Cardiac output was calculated as the product of heart rate and stroke volume. Total peripheral resistance was calculated as mean arterial pressure divided by cardiac output. Right middle cerebral artery blood velocity was measured via transcranial Doppler sonography using a 2 MHz probe (DWL USA, Inc., Germany, Europe) by the same research technician throughout all experimental visits. After a quality Doppler signal of middle cerebral artery blood velocity was obtained, the depth, gain, and location were recorded to be used for the second study visit. Middle cerebral artery conductance was calculated as middle cerebral artery blood velocity divided by mean arterial pressure.

Minute ventilation, tidal volume, and respiratory rate were determined using the breath by breath respiratory analysis feature of the data analysis software (AcqKnowledge 4.2, Goleta, CA, USA). Abhorrent breaths (e.g., sigh, breath hold, cough, etc.) were manually excluded and ventilation data are presented in body temperature and pressure, saturated (BTPS). The fraction of inspired CO_2_ was calculated as the inspired pressure of CO_2_ multiplied by the barometric pressure. The rate of CO_2_ production ($\dot{V}CO_{2}$) was calculated as minute ventilation multiplied by the fraction of expired CO_2_ minus the fraction of inspired CO_2_. Alveolar ventilation was calculated as the product of $\dot{V}CO_{2}$ and 863 divided by PETCO_2_ (West 2012), and dead space ventilation was calculated as minute ventilation minus alveolar ventilation. The alveolar ventilation to cardiac output ratio was calculated as an index of the alveolar ventilation to pulmonary perfusion ratio (Derion et al. 1992; Levitzky 2013).

### Rebreathing test

A rebreathing test was used to assess central chemoreceptor function (Read 1967; Rebuck 1976; MacKay et al. 2016). Subjects rebreathed 7% CO_2_ and 93% O_2_ for 3.5 min from a custom made 10 L anesthesia bag (Read 1967). The volume of the gas in the bag was equal to predicted vital capacity (Casio, 2018) plus 1 L. Data from the first 30 sec of the test were not used during analysis as this is when the CO_2_ pressure waveform is entering equilibrium.

Ventilatory sensitivity to CO_2_ was determined by plotting mean minute ventilation versus mean PETCO_2_ every 30 sec throughout the rebreathing test. Ventilatory sensitivity to CO_2_ data are reported as the slope of the linear regression line of minute ventilation versus PETCO_2_. The ventilatory threshold to CO_2_ is represented by the *x*‐intercept of the linear regression line of mean minute ventilation versus mean PETCO_2_. This value represents the theoretical PETCO_2_ value at which minute ventilation is 0 L/min (Read 1967). We also calculated the rate at which minute ventilation and PETCO_2_ increased over time throughout each rebreathing test. These data provide insight as to which variable (i.e., minute ventilation or PETCO_2_) is contributing to changes in ventilatory sensitivity to CO_2_. The increase in minute ventilation and PETCO_2_ over time were calculated as the change in minute ventilation and PETCO_2_, respectively, from the beginning to the end of the test, divided by the change in time (i.e., 3.5 min) (Fowle and Campbell 1964).

We calculated cerebrovascular reactivity to CO_2_ during the rebreathing test as an indicator of the sensitivity of middle cerebral artery blood velocity to changes in PETCO_2_ (MacKay et al. 2016). This was determined by plotting mean middle cerebral artery blood velocity versus mean PETCO_2_ every 30 sec throughout the rebreathing test. Cerebrovascular reactivity to CO_2_ data are reported as the slope of the linear regression line of middle cerebral artery blood velocity versus PETCO_2_ (MacKay et al. 2016).

### Experimental approach

The two experimental visits, in order, included: (1) a HOWI visit (HOWI) and (2) a matched hypercapnia visit performed in dry conditions (Dry + CO_2_). The HOWI visit always occurred first in order to establish the level of hypercapnia that was matched during Dry + CO_2_. The experimental visits did not occur on consecutive days and took place within a maximum of 6 days for all subjects. Subjects arrived at the laboratory having refrained from exercise, alcohol, and caffeine for 12 h, and food for 2 h for both experimental visits. Subjects also arrived to the laboratory euhydrated for both HOWI (urine specific gravity: 1.009 ± 0.005) and Dry + CO_2_ (urine specific gravity: 1.009 ± 0.007) visits. All data collection took place in a temperature controlled laboratory (24 ± 2°C, 41 ± 14% relative humidity) while subjects viewed a nonstimulating documentary and were encouraged to breathe normally. Following at least 10 min of seated rest, a baseline rebreathing test commenced. Upon completion of the baseline measurements, the subjects emptied their bladder and either entered the pool (HOWI) or continued seated rest (Dry + CO_2_) for 1 h. Over the next hour, a rebreathing test was performed at 10, 30, and 60 min. During HOWI, subjects were seated in thermoneutral water (35.0 ± 0.1°C) up to the neck. During Dry + CO_2_, small amounts of CO_2_ were added to the inspirate from a pre‐mixed gas tank (i.e., 13% CO_2_, 21% O_2_, and 66% N_2_) to match PETCO_2_ values that were obtained during HOWI. CO_2_ was added at the inspired side of a two‐way nonrebreathing valve (Hans Rudolph, Inc., Shawnee, KS, USA), such that the fraction of inspired CO_2_ induced hypercapnia. The flow of the added CO_2_ gas mixture was titrated to match the PETCO_2_ that occurred during the HOWI visit. After one hour of HOWI or Dry + CO_2_, subjects exited the pool or the addition of CO_2_ to the inspirate ceased. At this point, the subjects emptied their bladder and a rebreathing test commenced after 10 min of seated rest (i.e., post).

### Data and statistical analyses

Ventilatory data were captured at 62 Hz and hemodynamic data were obtained at 1 kHz by a data acquisition system (Biopac MP 150, Goleta, CA, USA). Data were stored on a personal computer for offline analyses. Data were assessed for approximation to a normal distribution, and an outlier analysis was performed. Outliers were identified and removed using the ROUT method (Motulsky and Brown 2006), in which the Q value, or the false discovery rate, was set conservatively (i.e., 0.1%) so that only definitive outliers were removed. Outliers were only removed from statistical analyses for ventilatory threshold to CO_2_ (*n* = 2). Following the outlier analysis, data were analyzed using a two‐way repeated measures ANOVA. If a significant interaction or main effect was found, the Holm‐Sidak multiple comparisons post hoc test was used to determine where differences existed. All data were analyzed using Prism software (Version 6, GraphPad Software Inc., La Jolla, CA, USA) and data are reported as mean ± SD. Significance was set a prior to *P* < 0.05 and exact *P*‐values are reported where possible.

## Results

### Body weight and urine loss

Reductions in body weight were not different following HOWI versus Dry + CO_2_ (0.68 ± 0.37 vs. 0.58 ± 0.35 kg, *P* = 0.125). However, urine loss was greater during HOWI versus Dry + CO_2_ (0.57 ± 0.38 vs. 0.44 ± 0.37 L, *P* = 0.046).

### Ventilation

**Figure 1:**
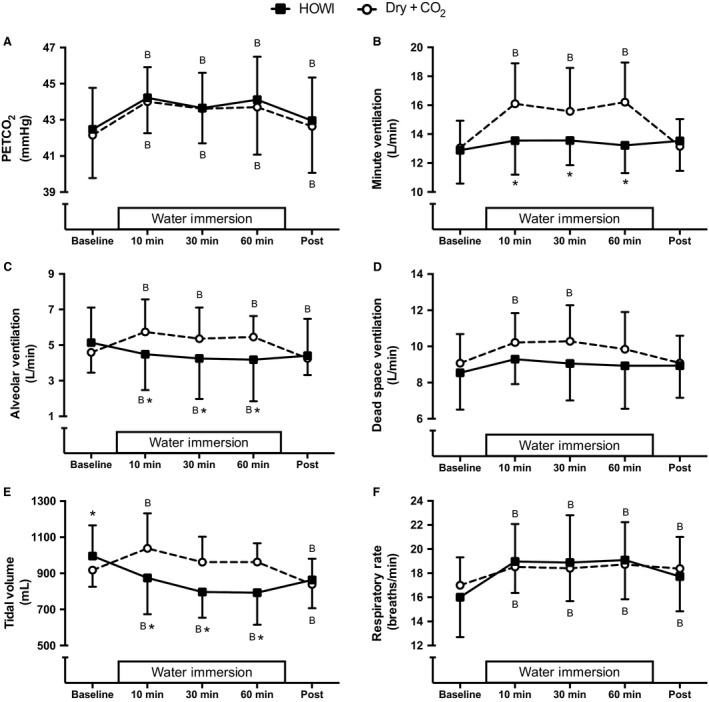
PETCO_2_ (A), minute ventilation (B), alveolar ventilation (C), dead space ventilation (D), tidal volume (E), and respiratory rate (F) at baseline, 10, 30, 60 min, and post HOWI and Dry + CO_2_. Water immersion only occurred during the HOWI visit. Values are mean ± SD. * = different from Dry + CO_2_, *P* < 0.050. B = different from baseline, *P* < 0.050.

PETCO_2_ (Fig. 1A) was greater than baseline during HOWI and Dry + CO_2_ at 10 min (*P* < 0.001), 30 min (*P* < 0.001), 60 min (*P* < 0.001), and post (*P* = 0.013) and was successfully matched between conditions so that there was not a condition effect (condition main effect: *P* = 0.494) or an interaction effect (interaction main effect: *P* = 0.674). Minute ventilation (Fig. 1B) and alveolar ventilation (Fig. 1C) were lower during HOWI versus Dry + CO_2_ at 10 min (*P* ≤ 0.001), 30 min (*P* ≤ 0.003), and 60 min (*P* ≤ 0.001). Dead space ventilation (Fig. 1D) was greater than baseline during Dry + CO_2_ at 10 min (*P* = 0.011) and 30 min (*P* = 0.009) but was not different during HOWI versus Dry + CO_2_ at any time point (condition main effect: *P* = 0.193). Tidal volume (Fig. 1E) was greater during HOWI versus Dry + CO_2_ at baseline (*P* = 0.030) and lower during HOWI versus Dry + CO_2_ at 10 min (*P* < 0.001), 30 min (*P* < 0.001), and 60 min (*P* < 0.001). Respiratory rate (Fig. 1F) was greater than baseline throughout HOWI (*P* ≤ 0.003) and Dry + CO_2_ (*P* ≤ 0.026) and was not different between HOWI and Dry + CO_2_ (condition main effect: *P* = 0.831).

### Hemodynamics

**Figure 2:**
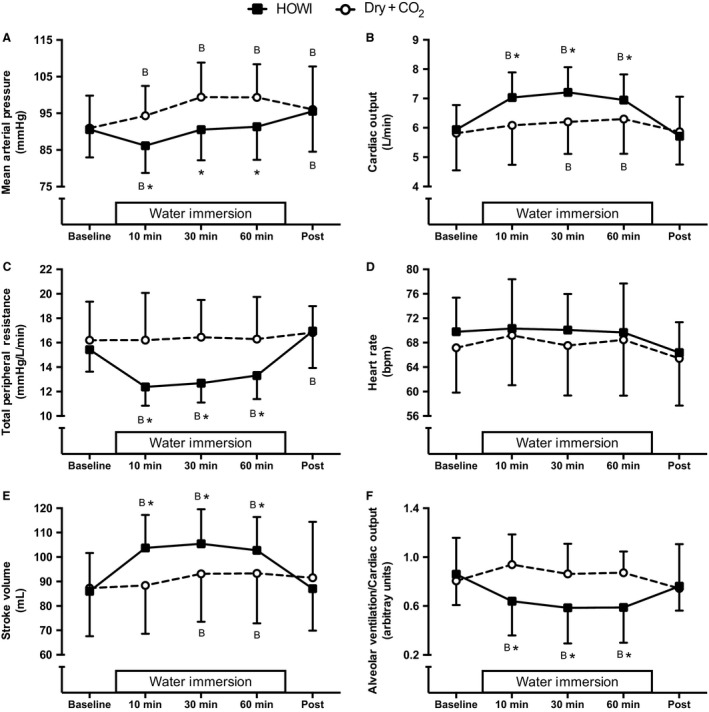
Mean arterial pressure (A), cardiac output (B), total peripheral resistance (C), heart rate (D), stroke volume (E), and alveolar ventilation to cardiac output ratio (F) at baseline, 10, 30, 60 min, and post HOWI and Dry + CO_2_. Water immersion only occurred during the HOWI visit. Values are mean ± SD. * = different from Dry + CO_2_, *P* < 0.050. B = different from baseline, *P* < 0.050.

Mean arterial pressure (Fig. 2A) was lower during HOWI versus Dry + CO_2_ at 10 min (*P* < 0.001), 30 min (*P* < 0.001), and 60 min (*P* < 0.001). Meanwhile, cardiac output (Fig. 2B) was greater than baseline during HOWI at 10 min (*P* < 0.001), 30 min (*P* < 0.001), and 60 min (*P* < 0.001), while total peripheral resistance (Fig. 2C) was lower than baseline during HOWI at 10 min (*P* < 0.001), 30 min (*P* < 0.001), and 60 min (*P* < 0.001). Cardiac output was greater during HOWI versus Dry + CO_2_ at 10 min (*P* < 0.001), 30 min (*P* < 0.001), and 60 min (*P* < 0.001), while total peripheral resistance was lower during HOWI versus Dry + CO_2_ at 10 min (*P* < 0.001), 30 min (*P* < 0.001), and 60 min (*P* < 0.001). Heart rate (Fig. 2D) was not different across time during HOWI (*P* ≥ 0.105) or Dry + CO_2_ (*P* ≥ 0.553) or between conditions (condition main effect: *P* = 0.419). Stroke volume was greater during HOWI versus Dry + CO_2_ at 10 min (*P* < 0.001), 30 min (*P* < 0.001), and 60 min (*P* < 0.001). The alveolar ventilation to cardiac output ratio (Fig. 2F) was less than baseline during HOWI at 10 min (*P* < 0.001), 30 min (*P* < 0.001), and 60 min (*P* < 0.001) and was lower during HOWI versus Dry + CO_2_ at 10 min (*P* < 0.001), 30 min (*P* < 0.001), and 60 min (*P* < 0.001).

### Cerebral hemodynamics

**Figure 3:**
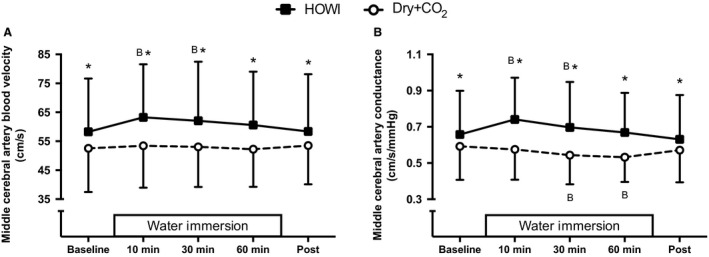
Middle cerebral artery blood velocity (A) and middle cerebral artery conductance (B) at baseline, 10, 30, 60 min, and post HOWI and Dry + CO_2_. Water immersion only occurred during the HOWI visit. Values are mean ± SD. * = different from Dry + CO_2_, *P* < 0.050. B =  different from baseline, *P* < 0.050.

Middle cerebral artery blood velocity was greater than baseline during HOWI at 10 min (*P* < 0.001) and 30 min (*P* = 0.004). Middle cerebral artery conductance (Fig. 3B) was greater than baseline during HOWI at 10 min (*P* < 0.001) and 30 min (*P* = 0.025), while it was less than baseline during Dry + CO_2_ at 30 min (*P* = 0.004) and 60 min (*P* < 0.001).

### Rebreathing test

**Figure 4:**
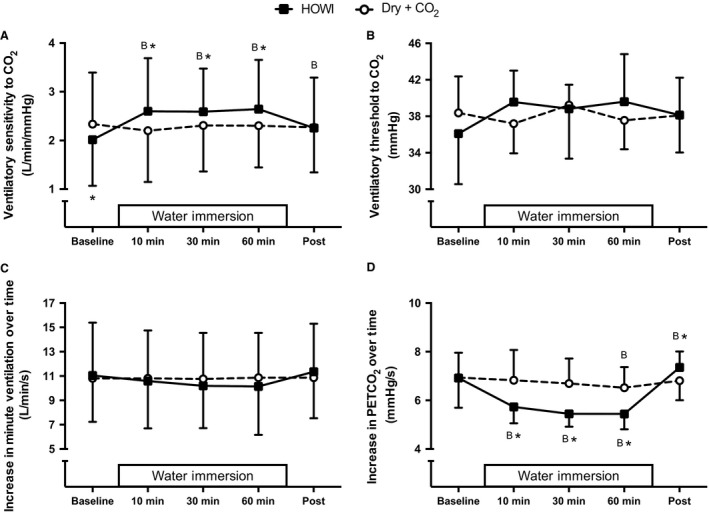
Ventilatory sensitivity to CO_2_ (A), ventilatory threshold to CO_2_ (B), increase in minute ventilation over time during the rebreathing test (C), and increase in PETCO_2_ over time during the rebreathing test (D) at baseline, 10, 30, 60 min, and post HOWI or Dry + CO_2_. Water immersion only occurred during the HOWI visit. Values are mean ± SD. * = different from Dry + CO_2_, *P* < 0.050. B = different from baseline, *P* < 0.050.

Ventilatory sensitivity to CO_2_ (Fig. 4A) was greater than baseline during HOWI at 10 min (*P* < 0.001), 30 min (*P* < 0.001), 60 min (*P* < 0.001), and post (*P* = 0.048), while ventilatory sensitivity to CO_2_ was not different than baseline throughout Dry + CO_2_ (*P* ≥ 0.702). Moreover, ventilatory sensitivity to CO_2_ was higher during HOWI versus Dry + CO_2_ at 10 min (*P* = 0.007), 30 min (*P* = 0.040), and 60 min (*P* = 0.025). There was not a time effect (time main effect: *P* ≥ 0.203), condition effect (condition main effect: *P* ≥ 0.694), or interaction effect (interaction main effect: *P* ≥ 0.054) for ventilatory threshold to CO_2_ (Fig. 4B) or the increase in minute ventilation over time (Fig. 4C). However, the increase in PETCO_2_ over time (Fig. 4D) was lower during HOWI versus Dry + CO_2_ at 10 min (*P* < 0.001), 30 min (*P* < 0.001), and 60 min (*P* < 0.001), and was greater during HOWI versus Dry + CO_2_ at post (*P* = 0.003).

**Figure 5:**
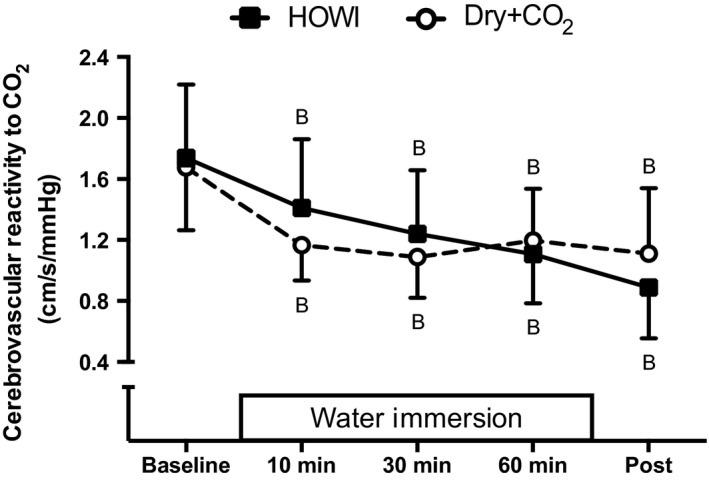
Cerebrovascular reactivity to CO_2_ at baseline, 10, 30, 60 min, and post HOWI or Dry + CO_2_. Water immersion only occurred during the HOWI visit. Values are mean ± SD. B = different from baseline, *P* < 0.050.

Cerebrovascular reactivity to CO_2_ (Fig. 5) was less than baseline throughout HOWI (*P* < 0.001) and Dry + CO_2_ (*P* < 0.001) but was not different between conditions (condition main effect: *P* = 0.777).

## Discussion

The novel findings of our investigation are despite a greater minute ventilation, ventilatory sensitivity to CO_2_ was lower during dry conditions when breathing a hypercapnic gas to match the hypercapnia that occurs during HOWI when compared to HOWI. Moreover, cerebral perfusion was lower during dry conditions while breathing a hypercapnic gas to match the hypercapnia that occurs during HOWI when compared to HOWI, while cerebrovascular reactivity to CO_2_ was not different between conditions. Thus, although acute hypercapnia increases minute ventilation, it does not augment ventilatory sensitivity to CO_2_ or cerebral blood flow and it lessens cerebrovascular reactivity to CO_2_. Thus, augmented ventilatory sensitivity to CO_2_ and cerebral perfusion during HOWI appear to be a function of the combined effects of water immersion and not elevated PETCO_2_ alone.

### Ventilation

Acute hypercapnia was induced during Dry + CO_2_ by adding small amounts of CO_2_ to the inspirate of the breathing apparatus. Thus, inspired CO_2_ levels were greater during Dry + CO_2_ versus HOWI by design. Increasing PETCO_2_ by raising inspired CO_2_ (i.e., end tidal forcing) has been used to investigate the effects of acute hypercapnia (Slessarev et al. 2007; Koehle et al. 2009; Mark et al. 2010). It is currently unclear if the differences in inspired CO_2_ contributed to the different ventilatory and hemodynamic responses that we observed in spite of tightly matching the PETCO_2_ values between conditions. In this context, failure of CO_2_ scrubbers increase inspired CO_2_ during underwater diving while using a rebreather system (Barlow and Macintosh 1944). Therefore, adding CO_2_ to the inspirate is a valid approach to study various underwater diving scenarios. Nonetheless, future investigations should examine if other effects of water immersion (i.e., work of breathing, central hypervolemia, etc.) contribute to increase PETCO_2_ during water immersion.

During Dry + CO_2_, we accurately matched the increase in PETCO_2_ that occurred during HOWI. This small, but significant, increase in PETCO_2_ allowed us to investigate the effect of acute hypercapnia alone on the ventilatory sensitivity to CO_2_ and cerebral perfusion. Despite that minute ventilation was unchanged throughout HOWI, the increase in PETCO_2_ during Dry + CO_2_ stimulated the chemoreceptors and increased minute ventilation. Similarly, alveolar ventilation was lower during HOWI versus Dry + CO_2_. Previous findings indicate that a reduced alveolar ventilation may contribute to hypercapnia during hyperbaria (Salzano et al. 1984). To our knowledge, our investigation is the first to demonstrate alveolar hypoventilation during HOWI, as this has only previously been observed during investigations at depth (Salzano et al. 1984; Mummery et al. 2003; Cherry et al. 2009). The concomitant increases in ventilation and PETCO_2_ during Dry + CO_2_ suggest a shift of the respiratory operating point (i.e., minute ventilation vs. PETCO_2_) (Miyamoto et al. 2014). Similarly, thermoneutral HOWI induces a shift of the respiratory operating point, and this is in agreement with previous investigations (Miyamoto et al. 2014; Sackett et al. 2017, 2018). The chemoreflex feedback system can be divided into two subsystems: i) the central controller and ii) the peripheral plant (Berger et al. 1977; Miyamoto et al. 2014). The central controller is described as the relation between minute ventilation and arterial CO_2_ pressure, such that minute ventilation increases linearly as a function of arterial CO_2_ pressure (i.e., ventilatory sensitivity to CO_2_) (Berger et al. 1977). On the other hand, the peripheral plant is described as the relation between arterial CO_2_ pressure and minute ventilation, such that arterial CO_2_ pressure decreases asymptotically as a function of minute ventilation (Berger et al. 1977). In this context, a shift of the respiratory operating point appears to be driven by the central controller during HOWI, while it is driven by the peripheral plant during Dry + CO_2_ (Ogoh et al. 2008; Miyamoto et al. 2014). However, the mechanisms underpinning these discrepancies are not clear. It has also been proposed that an increase in dead space ventilation might contribute to hypercapnia during water immersion (Salzano et al. 1984; Mummery et al. 2003; Cherry et al. 2009). Our data do not indicate an increase in dead space ventilation during HOWI despite a decrease in tidal volume and an increase in respiratory rate during HOWI. These changes in breathing pattern are thought to be a result of increased respiratory work during water immersion (Salzano et al. 1984; Moon et al. 2009; Ray et al. 2010). Such increases in respiratory work during HOWI may have prevented elevations in minute ventilation. To this end, an increase in tidal volume may be possible in the absence of increased respiratory work (i.e., Dry + CO_2_) (Sackett et al. 2017, 2018). This was evident during Dry + CO_2_ as increases in tidal volume and respiratory rate were achieved in the absence of increased respiratory work. Further investigations should elucidate if an increase in respiratory work causes a decrease in tidal volume during HOWI and subsequently contributes to hypercapnia during HOWI.

### Hemodynamics

Mean arterial pressure increased throughout Dry + CO_2_ but not during the HOWI visit until post HOWI and it was lower during HOWI versus Dry + CO_2_ throughout water immersion. HOWI induced increases in cardiac output that were accompanied by decreases in total peripheral resistance, while Dry + CO_2_ induced increases in cardiac output, without a change in total peripheral resistance. The reduction in total peripheral resistance during HOWI is most likely a result of the water‐skin temperature gradient (Pendergast et al. 2015; Sackett et al. 2017, 2018) and attenuated sympathetic nerve activity during water immersion (Mano et al. 1985; Miwa et al. 1996). It is not clear why Dry + CO_2_ did not appear to induce vasodilation (i.e., a reduction in total peripheral resistance). The effects of acute hypercapnia during dry conditions on vascular responses and sympathetic nerve activity (i.e., neurovascular transduction) are currently not known. The increase in stroke volume during HOWI and Dry + CO_2_ was the primary cause for the rise in cardiac output in both conditions. While this typically occurs during HOWI due to the hydrostatic pressure induced central hypervolemia and elevated venous return (Arborelius et al. 1972), it appears that an increase in arterial CO_2_ alone augments stroke volume and results in a modest increase in cardiac output. It is possible that increases in minute ventilation during Dry + CO_2_ enhanced venous return and therefore stroke volume through the thoracic pump (Taylor and Groeller 2008). Moreover, despite previous findings (Sackett et al. 2018), we found a reduction in the alveolar ventilation to cardiac output ratio during HOWI, which might contribute to increases in PETCO_2_ by impairing gas exchange (Derion et al. 1992; Moon et al. 2009). Previous work has demonstrated that minute ventilation is unchanged (Sackett et al. 2017, 2018) or reduced (Cherry et al. 2009) during water immersion. If we assume the typical rise in central blood volume during water immersion (Arborelius et al. 1972), these prior findings also suggest that a reduced alveolar ventilation to cardiac output ratio occurs during HOWI. Along these lines, pulmonary blood flow is augmented during water immersion, which may contribute to increases in CO_2_ pressure (Moon et al. 2009). Although unlikely, we cannot confirm that pulmonary blood flow was not elevated during Dry + CO_2_.

### Cerebral hemodynamics

Previous investigations indicate that middle cerebral artery blood velocity is augmented during HOWI (Carter et al. 2014). Middle cerebral artery blood velocity reflects total cerebral blood flow and is correlated with changes in PETCO_2_ (Hida et al. 1996; Garbin et al. 1997; Barrett et al. 2001). Our data indicate that middle cerebral artery blood velocity is not altered during Dry + CO_2_ and lower during Dry + CO_2_ versus HOWI, which suggests that an increase in middle cerebral artery blood velocity during HOWI is due to the combined effects of water immersion (i.e., elevated PETCO_2_, central hypervolemia, increased work of breathing, etc.). Carter et al. suggested that an increase in middle cerebral artery blood velocity during water immersion is due to increases in blood pressure (Carter et al. 2014). However, they used a colder water temperature than we did (~30°C vs. ~35°C), which would likely cause differences in total peripheral resistance between studies. Thermoneutral water immersion (~35°C) reduces muscle sympathetic activity in the tibial nerve (Mano et al. 1985; Miwa et al. 1996), which likely contributes to increases in systemic blood flow occurring secondary to reductions in peripheral vascular resistance (Arborelius et al. 1972). However, it is currently unknown how cerebral sympathetic and parasympathetic nerves (Edvinsson et al. 1993) contribute to cerebral blood flow during water immersion.

### Rebreathing test

Similar to previous findings (Sackett et al. 2018), our data indicate that ventilatory sensitivity to CO_2_ was augmented during HOWI and greater than during Dry + CO_2_, since ventilatory sensitivity to CO_2_ was not altered during Dry + CO_2_. Thus, elevated PETCO_2_ alone does not appear to contribute to the elevated ventilatory sensitivity to CO_2_ during HOWI. The ventilatory threshold to CO_2_ represents the minimum PETCO_2_ that causes activation of the central chemoreceptors (Read 1967). To this end, a rightward shift in the ventilatory threshold to CO_2_ indicates a higher PETCO_2_ for the same minute ventilation and could contribute to increases in ventilatory sensitivity to CO_2_, as previous work indicates (Sackett et al. 2018). However, we did not observe a rightward shift in the ventilatory threshold to CO_2_ during either visit in the current investigation. The reason for these discrepant findings are not inherently clear. Previous findings indicate that the rate of rise of PETCO_2_ during a rebreathing test is attenuated during water immersion (Chang and Lundgren 1995; Sackett et al. 2018) which is indicative of an increase in the CO_2_ storage capacity of the body (Fowle and Campbell 1964). Chang and Lundgren suggested that an elevated tissue perfusion during HOWI contributes to a greater CO_2_ redistribution to the tissues (i.e., muscle and fat) and may be related to the increase in PETCO_2_ (Chang and Lundgren 1995). The present data also indicate that a slower increase in PETCO_2_ over time occurs during HOWI. Thus, it appears that a slower increase in PETCO_2_ over time might contribute to increases in ventilatory sensitivity to CO_2_ during HOWI. It is currently unclear why PETCO_2_ increases at a slower rate during HOWI.

We found that cerebrovascular reactivity to CO_2_ was lower than baseline during both HOWI and Dry + CO_2_ but it was not different between conditions. These data indicate that increases in PETCO_2_, as a result of HOWI or breathing hypercapnic gas, blunts cerebrovascular reactivity to CO_2_. Previous findings indicate that minor reductions in cerebral blood flow attenuate cerebrovascular reactivity to CO_2_ and increase ventilatory sensitivity to CO_2_, while further reductions in cerebral blood flow cause a greater decrease in cerebrovascular reactivity to CO_2_ (Chapman et al. 1979). Our data indicate that reduced cerebrovascular reactivity to CO_2_ during HOWI may increase cerebral perfusion and that this occurs at the same time that ventilatory sensitivity to CO_2_ is elevated. It is unlikely, however, that these changes in cerebrovascular reactivity to CO_2_ associated with acute hypercapnia are causally mediating changes in ventilatory sensitivity to CO_2_ because decreases in cerebrovascular reactivity to CO_2_ were not accompanied by increases in ventilatory sensitivity to CO_2_ during Dry + CO_2_. It should be noted that rebreathing tests are considered ‘ventilatory independent’, such that changes in ventilation during rebreathing do not appear to be dependent on changes in cerebrovascular reactivity to CO_2_ (Ainslie and Duffin 2009). Although this effect is often negligible (Pandit et al. 2007), future investigations using steady state methods are required to elucidate causation between cerebrovascular reactivity to CO_2_ and ventilatory sensitivity to CO_2_, especially during HOWI.

### Perspectives

PETCO_2_ increases during water immersion despite augmented ventilatory sensitivity to CO_2_ (Sackett et al. 2018). Moreover, an elevated PETCO_2_ increases an underwater diver's risk for several hypercapnia related symptoms. These symptoms include breathlessness, headaches, and dizziness and enhance the likelihood of CO_2_ toxicity and unexpected loss of consciousness while diving (Fothergill et al. 1998). Collectively, these symptoms and conditions can lead to life‐threatening situations in both recreational and working underwater divers. Lundgren demonstrated that the static lung load during HOWI mimics that of underwater diving at depth in the upright position while using an underwater breathing apparatus (Lundgren and MIller 1999). However, previous reports indicate dose dependent elevations in end tidal and/or arterial CO_2_ pressures with depth and exercise (Lanphier and Bookspan 1999; Cherry et al. 2009). It is therefore possible that water immersion at depth or during exercise may further enhance end tidal and/or arterial CO_2_ pressures and contribute to alterations in ventilatory sensitivity to CO_2_ and cerebrovascular reactivity to CO_2_. Furthermore, water immersion induces similar ventilatory and hemodynamic alterations that occur during microgravity, including an elevated PETCO_2_ (Nagatomo et al. 2014; Watenpaugh 2016). To this end, immersion is often used as a model of microgravity (Watenpaugh 2016). CO_2_ pressure is elevated during space flight as a result of increases in the ambient partial pressure of CO_2_ in the spacecraft (Law et al. 2010). Consequently, flight crew members commonly report symptoms such as headaches and nausea, which are associated with hypercapnia (Law et al. 2010). Previous reports indicate that these symptoms may hinder the mission outcomes by contributing to fatigue, lethargy, and confusion (Carr 2006). Therefore, identifying the mechanisms that underpin the elevated CO_2_ pressure and changes in ventilatory sensitivity and cerebrovascular reactivity are important to develop countermeasures to prevent increases in CO_2_ pressure during water immersion and space flight. Current evidence indicates that respiratory muscle training normalizes ventilatory sensitivity to CO_2_ during dry conditions in subjects with low or high baseline ventilatory sensitivity to CO_2_ (Pendergast et al. 2006) and could therefore be an intervention to reduce the risk of CO_2_ toxicity.

### Considerations

Our investigation has several considerations worth noting. First, HOWI induced a relatively small increase in PETCO_2_. This small increase in PETCO_2_ during HOWI may not be considered clinically significant (Moloney et al. 2001). However, it was apparent that the increases in PETCO_2_ were sensed by the chemoreceptors during Dry + CO_2_, evidenced by the rise in minute ventilation. Along these lines, we used PETCO_2_ as a marker of arterial CO_2_ pressure since previous reports demonstrate that PETCO_2_ and arterial CO_2_ pressure are not significantly different during water immersion (Dunworth et al. 2017). However, PETCO_2_ may underestimate arterial CO_2_ pressure during instances of increased dead space (i.e., water immersion) (Liu et al. 1995; Williams and Babb 1997). Thus, using PETCO_2_ as a surrogate of arterial CO_2_ pressure is a conservative approach, such that calculated differences in dead space ventilation and alveolar ventilation to cardiac output ratio would be exacerbated if we were to observe a higher value for arterial CO_2_ pressure via direct measurements. It is also possible that increases in PETCO_2_ during water immersion may be caused by other factors such as, alveolar ventilation to perfusion mismatching. Second, we recruited a convenience sample for the current investigation and therefore the subjects were not restricted to divers or nondivers. Regular underwater divers have attenuated ventilatory sensitivity to CO_2_ compared to nondivers during dry conditions (Pendergast et al. 2006; Earing et al. 2014). To this end, several subjects were certified underwater divers (*n* = 5) but only a few reported to be regular divers (*n* = 2). Despite this, all subjects who were underwater divers had not been diving within 1 month of experimental testing. However, it is currently not known if divers and nondivers have similar ventilatory and cerebrovascular control during water immersion. Moreover, we did not determine if subjects had an undiagnosed patent foramen ovale. Subjects with a patent foramen ovale may have impaired gas exchange (Lovering et al. 2011), which could contribute to elevated PETCO_2_ during water immersion. Third, subjects were seated upright throughout both experimental visits. While this was part of our experimental design in an attempt to eliminate any effects of posture, it should be noted that venous pooling may have occurred in the lower extremities during Dry + CO_2_ but not during HOWI. Thus, we cannot be certain that venous pooling in the legs did not contribute to our findings. Fourth, we found that ventilatory sensitivity to CO_2_ was greater during HOWI versus Dry + CO_2_ at baseline. The reproducibility of ventilatory sensitivity to CO_2_ has been shown to be good across a few hours and days (Scamman and Ghoneim 1983; Sullivan and Yu 1984). It is unclear why we found this baseline difference as we controlled for factors that might contribute to day to day variation in ventilatory sensitivity to CO_2_ (i.e., fasting for 2 h, abstaining from exercise, alcohol, and caffeine for 12 h, and being euhydrated) and both visits were performed within one week. Finally, we used transcranial Doppler to measure middle cerebral artery blood velocity during our investigation. Although recent evidence indicates that middle cerebral artery blood velocity is indicative of total cerebral blood flow (Hida et al. 1996; Garbin et al. 1997; Barrett et al. 2001), it is not known if this is the case during water immersion. In this regard, we were unable to measure changes in cerebral vessel diameter, which may have occurred during the experimental visits due to hypercapnia and/or HOWI (Carter et al. 2014).

## Conclusions

In sum, although minute ventilation is greater, ventilatory sensitivity to CO_2_ is lower during dry conditions while breathing hypercapnic gas to match the elevated PETCO_2_ that occurs during HOWI when compared to HOWI. Meanwhile, cerebral perfusion is lower, while cerebrovascular reactivity to CO_2_ is not different during dry conditions while breathing a hypercapnic gas to match the elevated PETCO_2_ that occurs during HOWI when compared to HOWI. Augmented ventilatory sensitivity to CO_2_ and cerebral perfusion during HOWI appears to be a function of the integrative physiological changes that occur during water immersion and not elevated PETCO_2_ (i.e., acute hypercapnia) alone. Therefore, it appears that small increases in PETCO_2_ are not physiologically important with regards to modifying ventilatory sensitivity to CO_2_ and cerebral perfusion during HOWI.

## Conflict of Interest

There are no competing interests to report.
